# Supplementary material for: What does it take to learn the rules of RNA base pairing? A lot less than you may think
Source: bioRxiv. 2025 Aug 2:2025.07.31.668042. Preprint. [Version 1] doi: 10.1101/2025.07.31.668042 (PMC12324431; doi:10.1101/2025.07.31.668042)
Supplement: Supplement 1 [file media-1.gz › supplemental_material/src/rscape_v2.6.0.tar.gz!/rscape_v2.6.0/data/power/Rfam.15.0.seed.3D.power.subs.pdf]

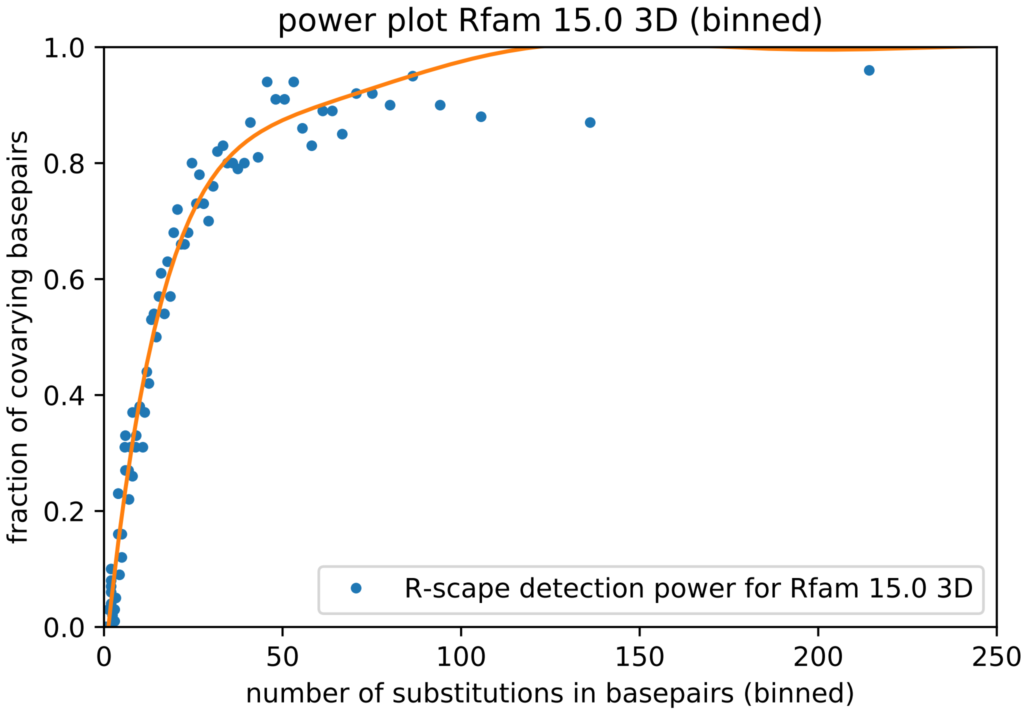

power plot Rfam 15.0 3D (binned)
fraction of covarying basepairs
number of substitutions in basepairs (binned)
R-scape detection power for Rfam 15.0 3D
0.0
0.2
0.4
0.6
0.8
1.0
0
50
100
150
200
250
